# Involvement of the Peripheral Nervous System in Episodic Ataxias

**DOI:** 10.3390/biomedicines8110448

**Published:** 2020-10-22

**Authors:** Wojciech Koźmiński, Joanna Pera

**Affiliations:** 1 Department of Neurology, University Hospital, ul. Jakubowskiego 2, 30-688 Krakow, Poland; wojciechmed@gmail.com; 2 Department of Neurology, Jagiellonian University Medical College, ul. Botaniczna 3, 31-503 Krakow, Poland

**Keywords:** episodic ataxia, peripheral nervous system, channels

## Abstract

Episodic ataxias comprise a group of inherited disorders, which have a common hallmark—transient attacks of ataxia. The genetic background is heterogeneous and the causative genes are not always identified. Furthermore, the clinical presentation, including intraictal and interictal symptoms, as well as the retention and progression of neurological deficits, is heterogeneous. Spells of ataxia can be accompanied by other symptoms—mostly from the central nervous system. However, in some of episodic ataxias involvement of peripheral nervous system is a part of typical clinical picture. This review intends to provide an insight into involvement of peripheral nervous system in episodic ataxias.

## 1. Introduction

Episodic ataxias (EAs, OMIM: phenotypic series PS160120, hereditary episodic ataxia ORPHA:211062) are a group of inherited disorders with a common denominator in the form of transient attacks of incoordination. However, the duration and triggers of these attacks vary between different types of EAs. Moreover, the neurological symptoms present during attacks and/or between them are different. In some patients, the resolution of the neurological deficits is incomplete, and these deficits increase with the duration of the disease. This heterogeneity can be observed even within families with the same mutation and factors (including genetic and environmental) that modify the clinical presentation remain largely unknown. In some of the EAs symptoms from the peripheral nervous system (PNS) are part of the clinics.

The catalogue of EAs seems to be incomplete. Causative genes were identified in some of them, but in some EAs we know only the approximate gene location. The most common types are EA1 and EA2. Their causative genes encode the alpha subunits of potassium K_v_1.1 and calcium Ca_v_2.1 voltage-gated channel, respectively. These two EAs remain relatively best characterized on the molecular level when compared to the rest of EAs. However, even in their case, the genotype–phenotype correlation is unknown. So far, all described EAs have an autosomal dominant pattern of inheritance.

This review aims to present an overview of PNS involvement in EAs and the underlying pathophysiology. A brief summary presents Table 1.

## 2. Episodic Ataxia Type 1

The causative gene for EA1 (OMIM: #160120, ORPHA:211062) is KCNA1 on chromosome 12p13.32. This ataxia is also known as episodic ataxia with myokymia, paroxysmal ataxia with neuromyotonia, and hereditary myokymia with periodic ataxia. Its clinical presentation varies between and even within families. Its onset is typically in childhood or early adolescence with brief attacks lasting seconds to minutes of different frequency—from about a dozen per day up to less than once a month. These attacks can be provoked by exertion, abrupt postural change, stress, emotions, fatigue, vestibular stimulation, caffeine, alcohol, ischemia, temperature changes, food rich in salt, bitter oranges, or chocolate. Attacks manifest in generalized ataxia with loss of balance, incoordination of hands, dysarthria, dizziness, blurry vision/diplopia, nausea, vomiting, diaphoresis, dyspnea, muscle weakness, twitching, stiffening, neuromyotonia, myokymia in the limbs, facial muscles (perioral, periorbital muscles), or hands (lateral finger movements). The phenotypic spectrum of EA1 is very broad with a substantial both inter- and intrafamilial variability without a defined genotype-phenotype correlation. In some patients, seizures of various type (tonic-clonic, partial) were reported. Symptoms from the PNS, i.e., myokymia and neuromyotonia can be also present between ataxic spells. Some patients present only peripheral symptoms without ataxia such as neuromyotonia alone or muscle cramps [1,2,3,4].

The gene product is potassium voltage-gated channel subfamily A member 1 (KCNA1), which forms K_v_1.1 channels. These are Shaker-related delayed-rectifier channels present both in the CNS and PNS, where the channel is especially enriched in basket cells in the cerebellum and in the juxtaparanodal regions as well as at branch points of myelinated motor axons, respectively [5,6,7,8,9,10]. Additionally, K_v_1.1 channels were found in visceral sensory neurons and this is associated with cardiorespiratory reflexes [11]. This channel is also present in the heart, retina, kidney, skeletal muscle, and pancreatic islet cells [12]. The encoded protein product is composed of six transmembrane segments (S1–S6): S1–S4 comprise a voltage sensor domain and S5–S6 form the central pore region; the P-loop linking S4 and S5 is important for ion selectivity; S4 segment possess several positively charged amino acids, which cause a conformational change upon membrane depolarization. This underlies the voltage-dependent channel activation—opening of the ion pore. Fast inactivation or closure during the continued depolarization proceeds through the occlusion of the ion pore by a cytoplasmic peptide. The functional channel is a tetramer. Wild-type K_v_1.1 subunits typically form homotetramers; however, they can co-assemble with mutated subunits (as present in EA1) or with K_v_1.2 and K_v_1.4 subunits. Both N- and C-termini are cytoplasmic. The N-terminus of the channel is associated with β-subunits that can modify the inactivation properties of the channel as well as affect expression levels [12,13,14]. The potassium channels that contain K_v_1.1 subunits are fast channels, which are mostly closed at resting membrane potential. They are characterized by a relatively low activation threshold and rapid activation kinetics, but are slower than fast Na^+^ channels—they are also called delayed rectifiers. The efflux of potassium ions is driven by the electrochemical trans-membrane gradient leads to the hyperpolarization of the membrane potential and limits neuronal excitability. In axons of myelinated nerves these channels are densely expressed in the juxtaparanodal region and with lower densities in the internode. Their activation reduces the resistance of the internodal membrane and limits axonal hyperexcitability after an action potential. Dysfunction of these channels leads to excessive excitability of neurons and to an enhanced duration of action potentials [4,15,16,17,18].

Most of the identified mutations causative for EA1 are missense mutations distributed throughout the gene; however, nonsense mutations are also known [1,4,7]. The functional consequences of *KCNA1* gene mutations identified in EA1 patients were investigated using various experimental settings. In many in vitro studies mutant subunits alone (R167M, V174F, I177N, F184C, C185W, T226A, T226M, T226R, V234F, R239S, A242P, F244C, P244H, F249I, I262T, T284M, R297S, F303V, F307I, G311S, E325D, S342I, E395D, V404I, I407M, V408A, F414C, R417stop, and V478A) or together with wild-type forms were expressed in *Xenopus laevis* oocytes or mammalian cells (RBL—rat basophilic leukemia; COS-1—immortalized monkey kidney epithelial cells; CHO—Chinese hamster ovary, HEK-293—human embryonic kidney cells, *Ltk*-mouse cells). In general, mutations lead to loss-of-function effects. When expressed on their own, several mutant subunits show a reduction in peak current amplitude in comparison to wild-type channels. However, this reduction varies among the different EA1 mutations: from an undetectable current amplitude (suggesting that the channel subunits are nonfunctional) to an amplitude indistinguishable from wild-type subunits. Moreover, a wide variety of alterations in voltage threshold and gaiting kinetics was observed: some mutations shift the activation threshold to more depolarized level, others increase or decrease the rate at which channels activate, inactivate, or deactivate [18,19,20,21,22,23,24,25,26,27,28,29]. Results of some studies revealed that some mutations can affect channel trafficking through the Golgi apparatus or targeting/anchoring correctly to the membrane [12,29,30,31]. In general, all these investigated mutations result in loss of K_v_1.1 channel function with a subsequent reduction of potassium efflux through the mutated channel. This seems to be responsible for the abnormal neuronal excitability present in EA1.

Animal models for EA1 include *Kcna1*-null mice, V408A-knock-in-mice, and a rat model—N-ethyl-N-nitrosourea-mutagenized rat. The *Kcna1*-deficient mice do not recapitulate the EA1 phenotype. There are no spontaneous discharges from phrenic-diaphragm muscle preparations ex vivo; however, at non-physiological low temperatures (−20 °C stimulus-induced repetitive discharges are present. Additionally, ex vivo studies on a carotid body showed that hypoxia causes a significant increase in sensory discharges. This indicates that functional consequences of KCNA1 mutations involve respiratory reflexes during hypoxia [11]. A knock-in model was generated by insertion of the heterozygous mutation V408A in one *Kcna1* allele. Administration of isoproterenol to evoke stress-fear responses, induces loss of motor coordination resembling EA1. Studies with *in vivo* preparations of lateral gastrocnemius nerve-muscle showed that mutant animals exhibit spontaneous myokymic discharges consisting of repeated singlets, dublets or multiplets, despite motor nerve axotomy. Moreover, spontaneous bursting activity or evoked by sciatic nerve stimulation, was exacerbated by muscle fatigue, ischemia, and low temperatures. These stressors increased also the amplitude of compound muscle potential. In case of ischemia and muscle fatigue there are some common events such as: lactic acid production, pH changes, ATP depletion, and release of potassium and inorganic phosphate. They might exacerbate the excitability of myelinated axons and trigger these bursting activity observed in mutant mice. Two-photon laser scanning microscopy from the motor nerve ex vivo revealed spontaneous abnormal Ca^2+^ signals present only in mutant animals. Altogether, these results support the hypothesis that motor nerve is a key player in generation of myokymic activity [15,32,33]. In the rat model (N-ethyl-N-nitrosourea-mutagenized rat, named autosomal dominant myokymia and seizures, ADMS) a missense mutation (S309T) is located in the voltage-sensor domain (S4). All homozygous rats die prematurely with a mean lifetime of 16 days and no homozygous rats survive beyond postnatal day 18. The heterozygous rats exhibit myokymia, neuromyotonia, and generalized tonic-clonic seizures. They also show cold stress-induced tremor, neuromyotonia, and motor incoordination. Muscle twitching is present from 6 weeks of age, and typically is characterized by coordinated muscle contraction of the eyelid, the neck, and the extremities. The number of twitches increases with age until 18 weeks, then decreases. Electromyography (EMG) reveals spontaneous myoclonic discharges, correlated with muscle twitching of the hind limbs. Also, fore limbs EMG shows rhythmic multiple discharges with an intraburst frequency of 7 Hz. The firing pattern is similar to that observed in human myokymia. The protein expression and trafficking is normal [32].

In human EA1 patients the presence of continuous interictal motor activity as a consequence of peripheral nerve hyperexcitability is one of the hallmarks. Neuromyotonia (mild to severe) is present in most patients and may be constant. Typically, facial and hand muscles are affected. Persistent neuromyotonia may lead to increased muscle tone (stiffness), muscle hypertrophy, and skeletal deformities (including postural abnormality in infants with flexion of the fingers, wrists, elbows, and knees) [2,3,25,34,35]. In consequence of the involuntary muscle activity, some patients acquire contractures of their Achilles tendons which may require surgery [36]. Myokymia is continuously present in EA1 patients—between attacks, it may be clinically evident or only detectable by EMG. Typically, there is a fine rippling in the periorbital and perioral muscles or lateral finger movements [2,3,37]. An abnormal continuous activity of respiratory muscles was reported as associated with paroxysmal dyspnea [38]. D’Adamo et al. recently provided a comprehensive summary of the clinical symptoms among EA1 patients [4].

EMG reveals a continuous spontaneous muscle fiber activity with rhythmic or arrhythmic singlets, duplets, or multiplets. Intensity of this activity may depend on the environmental temperature, i.e., it is increased by warm and decreased by cold temperatures. Myokymia is unaffected by pharmacological block at the proximal portion of peripheral nerves, but is reduced by distal nerve block and abolished by inhibition of neuromuscular transmission. That suggests that myokymia results from abnormal hyperactivity in the peripheral nerve [24,26,34,39,40,41]. Interestingly, Moghimi et al. reported that EMG of finger flexor after 10 years of follow-up did not change significantly over years [37].

Some abnormalities in neuromuscular transmission were also reported. In approximately half of investigated EA1 patients after a repetitive stimulation at 20 or 50 Hz a decrement of the amplitude of the first compound muscle action potential (CMAP) followed by an increment of the second CMAP amplitude was observed [42,43].

The routine sensory and motor nerve conduction study (NCS) is normal. However, nerve excitability studies show some changes characteristic for EA1. Namely, stimulation of the median nerve at the wrist with recording from surface electrodes on the abductor pollicis brevis revealed: a markedly increased superexcitability and late susceptibility, a shortened relative refractory period, and in threshold electrotonus the increase in excitability due to a depolarizing current. The current–threshold relationship suggested that fewer channels were open at the resting membrane. These changes are characteristic for reduced fast potassium channel conductance [44,45].

Brain MRI is normal in most patients. Routine laboratory test with electrolytes and CK levels within the normal range [43].

The frequency and severity of EA1 attacks can be attenuated with carbamazepine, which is probably the first-line treatment. Additionally, some patients benefit from other anti-epilectic medications such as phenytoin, lamotrigine, valproic acid, or some benzodiazepines. Also, positive effects of the carbonic anhydrase inhibitor acetalozamide can be observed in some of EA1 patients [1,2,4,46,47].

## 3. Episodic Ataxia Type 2

The causative gene for EA2 (OMIM: #108500, ORPHA:97) is *CACNA1A* on chromosome 19p13.13. This ataxia is also known as episodic ataxia with nystagmus, nystagmus-associated cerebellopathy, familial paroxysmal ataxia, and acetazolamide-responsive hereditary paroxysmal cerebellar ataxia. Its clinical presentation varies between and within families. Its onset is typically in childhood or early adolescence. In comparison to EA1, the duration of attacks is significantly longer. Attacks last hours to days with different frequency—from three to four per week up to once or twice per year. There are several triggers identified such as exercise, stress, emotions, heat, fever, caffeine, and alcohol. Attacks manifest in generalized ataxia, dysarthria, vertigo, diplopia, nausea, vomiting, dystonia, hemiplegia, and headaches. From the PNS myasthenic weakness even generalized can be present. In contrast to EA1 myokymia is absent. In addition to EA2, there are two more allelic disorders caused by *CACNA1A* mutations with autosomal dominant pattern of inheritance: familial hemiplegic migraine type 1 (FHM1) and spinocerebellar ataxia type 6 (SCA6). FHM1 is caused by gain-of-function mutations and SCA6 is caused by CAG expansion. However, there are overlapping phenotypes of these three disorders, which makes the clinical picture more complicated. In addition, in some patients with *CACNA1A*, loss-of-function mutations, a severe form of early infantile epileptic encephalopathy, was reported [2,3,48,49].

The gene product is calcium voltage-gated channel subunit alpha1 A (CACNA1A), which forms Ca_v_2.1 channels present both in the CNS and PNS (at the neuromuscular junction, NMJ) and playing a crucial role in neurotransmitter release. P/Q-type Ca_v_2.1 channels are heteromultimers composed at least of a principal α1 subunit and auxiliary β and α2δ subunits. The α1A subunit consists of four transmembrane repeats (I-IV), each with six transmembrane α-helices (S1–S6): S4 is the primary voltage-sensing element of the channel, S1–S3 form an aqueous conduit that enables passage of the S4 α-helix through the membrane; S5 and S6 line the channel conduction pore, and the extracellular segment linking the S5 and S6 helices (P-loop) contains highly conserved glutamate residue in all four repeats forming the selectivity filter [12,50,51]. P/Q channels are responsible for a large part of the calcium ions influx that triggers neurotransmitter release both in the peripheral and central nervous system, namely, voltage-gated calcium channels open in response to membrane depolarization to let calcium into the cell, where calcium serves as an important second messenger [12].

Most of the mutations causing EA2 phenotype result in a truncated protein product, which is rapidly degraded, and loss-of-function phenotype. Most of these mutations affects P-loop or S5 or S6 helices [2,52,53]. Functional studies using the whole cell patch method on COS7 cells (immortalized monkey kidney epithelial cells) expressing mutant calcium channels complexes showed diminished current density and the current amplitude comparing with the wild-type channels [54]. In studies on *Xenopus laevis* oocytes, the co-expression of mutant channels with wild-type channels resulted in a decrease in the Ca^2+^ current amplitude elicited by depolarization. A potential explanation of this phenomenon is an inability to form a fully functional channel pore, a degradation of normal channels triggered by their bounding with mutant proteins. Alternatively, there is a limited space at the plasma membrane for Ca_v_2.1 channels and normal and mutant channels compete against each other. Some mutations shift the activation potentials towards more positive [51].

Approximately 90% of Ca channels in NMJ are of the P/Q-type. Thus, loss-of-function mutations in *CACNA1A* may disturb neuromuscular transmission. In a mice model of EA2–heterozygous leaner Ca_v_2.1 mutant mice–a 25% reduction of acetylcholine (Ach) release at NMJ was found, and 10% nerve-stimulation evoked release [55]. It is worth noting that none of the loss-of-function Ca_v_2.1 mouse mutants fully modeled the alterations of NMJ transmission found in EA2 patients. Amplitude and quantal content of the nerve stimulation-evoked end plate potentials (EPPs) were reduced in homozygous Ca_v_2.1 -/-, la/la, and rol/rol mice, and also (to a lower extent) in heterozygous la/+ and rol/+ mice. In contrast, the EPP amplitude and quantal content were unaltered in tg/tg and Ca_v_2.1-/- mice. A compensatory upregulation of non-P/Q Ca^2+^ channels (mainly R-type) was present in the homozygous mouse mutants, with the notable exception of rolling. Compensatory mechanisms downstream of Ca^2+^ influx that make Ca^2+^ ions more efficient in triggering exocytosis were also present at the NMJ of most mutants, as revealed by, e.g., an increased micro EPP (mEPP) frequency in rol/rol and tg/tg mice. In general, the functional studies of the loss-of function Ca_v_2.1 mouse mutants revealed several secondary presynaptic and postsynaptic compensatory changes that may affect excitability and may or may not normalize neurotransmission depending on the mouse mutation and the synapse [56].

Human EA2 patients may present a generalized myasthenic weakness, which corresponds well with the presence and role of Ca_v_2.1 channels at the NMJ. In a case series published in Neurology, 6 out of 13 families with EA2 patients complain of generalized fluctuating weakness [57]. Also, single case reports described EA2 patients with generalized weakness during attacks, occasionally accompanied by areflexia, dysarthria, and dysphagia [58,59,60]. Moreover, within a four-generation Chinese family, some of the affected members presented with myokymia and limb weakness [61]. Additionally, there is a report about an EA2 patient in whom a 24-h Holter monitoring showed sinus bradycardia with marked sinus arrhythmia [60].

Routine sensory and motor NCS as well as EMG is often normal in EA2 patients. In some patients EMG showed subtle and non-specific sign of myopathic type without any clinical signs of myopathy or, despite no muscular activity present at rest, increased insertional activity suggesting hyperactivity of muscular fibers was recorded [62,63]. However, a focused examination of neuromuscular junction is likely to demonstrate some abnormalities in neuromuscular transmission [64]. Single fiber EMG (SFEMG), which is a technique to examine the end-plate jitter or single muscle fibers in response to voluntary activation or axonal stimulation may show signs of presynaptic failure such as jitter and blocking improved with increased stimulation. Both voluntarily activated SFEMG, which is generally used in adults, and axonal stimulation (used in children) revealed both jitters and blocking in EA2 patients, which improved with higher frequencies of stimulation, suggesting a presynaptic defect of neuromuscular transmission. Similar results were reported in Lambert–Eaton myasthenic syndrome and botulism. In gain-of-function mutations of *CACNA1A* SFEMG yields normal results [54,64]. The NCS performed in the mentioned female patient with sinus arrhythmia revealed axonal sensory neuropathy with the reduced sensory action potential (SNAP) amplitude in the median and ulnar nerves bilaterally and unrecordable responses from the sural and superficial peroneal nerves bilaterally. In addition, low-compound muscle action potential (CMAP) amplitude in both peroneal nerves was observed [60].

In vitro studies on muscle biopsies from patients with truncation mutations revealed a reduced amplitude and quantal content of the EPP elicit by nerve stimulation and reduced or zeroed EPP depression during 20-Hz stimulation, suggesting impaired neurotransmission mainly due to reduced probability of Ach release. Moreover, a significant contribution of N-type channels was revealed in the initiation of neuromuscular transmission as one of potentially compensatory mechanisms in response to a mutation affecting the P-type channels. However, upregulation of N-type channels at neuromuscular synapses did not fully restore normal synaptic transmission resulting in presynaptic deficiency of neuromuscular transmission [40,64].

Studies with 125I-α-bungarotoxin binding revealed normal number of acetylcholine receptors per end plate [64]. Electron microscopy of the NMJ showed variable diminution in size of the presynaptic terminals and mildly overdeveloped postsynaptic membranes in comparison to a normal postsynaptic terminal [40].

Nerve excitability studies performed on motor axons in the median nerve at attack-free times in EA2 patients revealed disturbed excitability of motor axons with changes that can be modelled in shunt conductance between the internodal axolemma and exterior. The features seen in patients include an increase in threshold, a shortened relative refractory period, and greater threshold changes to the changes in membrane potential seen in threshold electrotonus. These abnormalities are qualitatively similar to these in EA1. However, some measures differed between these two groups of patients: late subexcitability was normal in EA2, there was greater accommodation to depolarizing currents in EA1, and superexcitability was more increased in EA1. As suggested by the research, this pattern of excitability changes may result from nodal malformation in the neonatal period reflecting an indirect effect of abnormal calcium current fluxes during development [65].

Two EA2 patients (daughter and father) presented with ictal hyperhidrosis with acute hypothermia (35.5 °C) of the extremities, and interictal chronic diarrhea, which was imputed to the effect on acetylocholine release in gastrointestinal submucosal neurons that requires Ca_v_2.1 [66].

Brain MRI may reveal atrophy of the cerebellar vermis. Routine laboratory test with electrolytes and CK levels within the normal range.

Acetazolamide is effective in controlling or reducing the frequency and severity of EA2 attacks in approximately two-thirds of patients. Alternatively, 4-aminopyridine can be used. Also, there is a single report of positive effects of a combined use of acetazolamide and levetiracetam [2,3,4,46,47,48].

## 4. Episodic Ataxia Type 3

The causative gene for EA3 (OMIM: 606554, ORPHA:79135) is unidentified, so far. The associated locus is on chromosome 1q42.

Onset of symptoms is in adulthood with attacks lasting minutes up to 6 h. They may be provoked by movement. Attacks manifest in ataxia, vertigo, tinnitus, blurry vision/diplopia, and headache. From the PNS symptoms—myokymia may be present interictally [2,67].

Most patients respond to acetazolamide [2,3].

## 5. Episodic Ataxia Type 4

The genetic background of EA4 (OMIM: 606552, ORPHA:79136) remains unknown.

Onset of symptoms is in adulthood. Attacks last hours and no triggers have been identified so far. They manifest in ataxia, vertigo, and diplopia. Myokymia or other symptoms from the periphery have not been described [2]. It also known as periodic vestibulocerebellar ataxia (PATX). However, in the literature we can find the report by Steckley et al., who described a family with an AD disorder presenting with episodic ataxia, vertigo, and tinnitus, with interictally present myokymia, distinct than EA1, EA2, and a PATX and proposed to name that EA4 [68].

Typically, acetazolamide is ineffective and no specific pharmacotherapy is known [2,3].

## 6. Episodic Ataxia Type 5

The causative gene for EA5 (OMIM: 613855, ORPHA:211067) is calcium voltage-gated channel auxiliary subunit beta 4 (*CACNB4*) gene located on chromosome 2q23.2 and encoding voltage-dependent L-type calcium channel subunit beta-4 (CACNA4β4) protein. This subunit interacts directly with the C-terminus of α1 subunit (Ca_v_2.1) and participates in the modulation of calcium current amplitude, voltage dependence, and kinetics of activation and inactivation.

Onset is in adulthood. Typical attacks last hours but episodes lasting weeks were described. Identified triggering factors are: lack of sleep, fatigue, alcohol intake, copious meals, intercurrent infection, and psychological stress. Attacks manifest in ataxia and vertigo. One patient presented gradually progressive symptoms with unilateral numbness and weakness, headache, brief loss of consciousness with muscle relaxation followed by generalized weakness and incoordination. Symptoms from the PNS were not reported, so far. Brain MRI, CSF examination, caloric labyrinthine tests were normal [2,69].

Two allelic disorders are known: generalized epilepsy (C104F mutation) and juvenile myoclonic epilepsy (R482X) [2].

Administration of acetazolamide is effective in prophylaxis of EA5 attacks [2,3,69].

## 7. Episodic Ataxia Type 6

EA6 (OMIM: 612656, ORPHA:209967) is caused by mutations in solute carrier family 1 member 3 (*SLC1A3*) gene on chromosome 5p13.2. The gene product is excitatory amino acid transporter 1 (EAAT1) protein—a glutamate transporter expressed throughout the CNS.

Onset is in infancy and early childhood with attacks lasting hours to days, which may be provoked by fever or stress. Attacks manifest not only in ataxia (which can be present and progress interictally) but also in vertigo, seizures, hemiplegia, hemianopia, and migraine.

There are no peripheral symptoms [2].

Brain MRI may show mild cerebellar atrophy and during attacks a hyperintense signal in FLAIR sequence may be visible. Routine laboratory test with electrolytes and CK levels within the normal range. NCS and EMG is normal.

Treatment with acetazolamide is effective [2,3].

## 8. Episodic Ataxia Type 7

The causative gene for EA7 (OMIM: 611907, ORPHA:209970) is unidentified, so far. The associated locus is on chromosome 19q13.

Onset is the first–second decade of life. Attacks last hours to days and may be provoked by exertion or excitement. They present with ataxia, dysarthria, weakness, and vertigo in some patients. Symptoms from the PNS were not reported, so far [2,70].

Effective pharmacotherapy remains unknown [2,3].

## 9. Episodic Ataxia Type 8

The causative gene for EA8 (OMIM: 616055, ORPHA:401953) is mapped to chromosome 1p36.13-p34.3 and the suspected gene is ubiquitin protein ligase E3 component N-recognin 4 (*UBR4*) encoding E3 ubiquitin-protein ligase UBR4. UBR4 is known to interact with calmodulin, a Ca^2+^ regulating protein in the cytoplasm, and thus may be involved in the regulation of neuronal excitability [3].

Age of onset reported is 1 to 2 years. Attacks last minutes to hours and may be provoked by stress or tiredness. Attacks manifest in ataxia, dysarthria, nystagmus, myokymia, ocular twitching, and migraine [2,70].

So far, a positive response to clonazepam but not acetazolamide was observed [3].

## 10. Episodic Ataxia Type 9

According to the OMIM phenotypic series, EA9 (OMIM: #618924) is caused by heterozygous mutation in the SCN2A gene on chromosome 2q23. Furthermore, the allelic variants comprise the less severe phenotype of benign familial infantile seizure type 3 (BFIS3) and the more severe phenotype epileptic encephalopathy, early infantile, 11 (EIEE11). Despite wide range of clinical symptoms, the PNS involvement was not reported, so far.

In the report published in Annals of Clinical and Translational Neurology, Piarroux et al. described four patients with atactic attacks lasting minutes to days, with a childhood onset. This phenotype, the authors proposed to name EA9. The identified mutation was in *FGF14* gene. No symptoms from the periphery were reported [71].

In addition, there are a number of genetic disorders that can present with some EA-like phenotypes. Many of their causative genes encode proteins important for the ion transport (channels and proteins interacting with them, pumps, and transporters) [3,72]. Their detailed presentation is beyond the scope of this review.

## 11. Conclusions

In summary, the involvement of PNS is typical part of EA1—in the form of myokymia and neuromyotonia. Also, EA2 patients can present symptoms related to PNS involvement—as a consequence of disturbed NMJ transmission. As for the other rare types of EA—myokymia was also reported in EA3 and EA4 interictally and in EA8.

## Figures and Tables

**Table 1:** List of episodic ataxias (EAs).

Disease	OMIM	ORPHA	Gene/Locus	Inheritance	PNS Involvement
Episodic ataxia 1 (EA1)	160120	211062	*KCNA1*, 12p13.32	AD	Myokymia, neuromyotonia
Episodic ataxia 2 (EA2)	108500	97	*CACNA1A*, 19p13.13	AD	Myasthenic weakness can be generalized and fluctuating; SFEMG: jitter and blocking
Episodic ataxia 3 (EA3)	606554	79135	1q24	AD	Myokymia interictally
Episodic ataxia 4 (EA4)	606552	79136	(?)	AD	Myokymia interictally
Episodic ataxia 5 (EA5)	613855	211067	*CACNB4*, 2q23.2	AD	Not reported
Episodic ataxia 6 (EA6)	612656	209967	*SLC1A3*, 5p13.2	AD	Not reported
Episodic ataxia 7 (EA7)	611907	209970	19q13	AD	Not reported
Episodic ataxia 8 (EA8)	616055	401953	*UBR4*(?), 1p36.13-p.34.3	AD	Myokymia

## Abbreviations

EA	Episodic ataxia
PNS	Peripheral nervous system
NMJ	Neuromuscular junction
